# In vitro infection of human ocular tissues by SARS-CoV-2 lineage A isolates

**DOI:** 10.1186/s12886-022-02728-w

**Published:** 2022-12-30

**Authors:** Venkatramana D. Krishna, Heidi Roehrich, Declan C. Schroeder, Maxim C.-J. Cheeran, Ching Yuan, Joshua H. Hou

**Affiliations:** 1 Department of Veterinary Population Medicine, University of Minnesota, Minneapolis, MN USA; 2 Department of Ophthalmology and Visual Neurosciences, University of Minnesota, 420 Delaware St SE, MMC 493, Minneapolis, MN 55455 USA; 3 School of Biological Sciences, University of Reading, Reading, UK; 4 Lions Gift of Sight Eye Bank, St. Paul, MN USA

**Keywords:** SARS-CoV-2, COVID-19, Corneal epithelium, Corneal endothelium

## Abstract

**Background:**

The purpose of this study was: [1] to evaluate the infectivity of two SARS-CoV-2 lineage A variants on human ocular tissues in vitro*,* and [2] to evaluate the stability of SARS-CoV-2 lineage A variants in corneal preservation medium.

**Methods:**

Primary cultures of donor corneal, conjunctival, and limbal epithelium were inoculated with two lineage A, GISAID clade S isolates of SARS-CoV-2 (Hong Kong/VM20001061/2020, USA-WA1/2020), to evaluate the susceptibility of the ocular tissue to infection. Flat-mounted Descemet’s Stripping Automated Endothelial Keratoplasty (DSAEK) grafts were inoculated with SARS-CoV-2 to evaluate the susceptibility of the endothelium to infection. All inoculated samples were immunostained for SARS-CoV-2 nucleocapsid (N)-protein expression to confirm positive infection. SARS-CoV-2 Hong Kong was then inoculated into cornea preservation media (Life4°C, Numedis, Inc.). Inoculated media was stored at 4^o^C for 14 days and assayed over time for changes in infectious viral titers.

**Results:**

Corneal, conjunctival, and limbal epithelial cells all demonstrated susceptibility to infection by SARS-CoV-2 lineage A variants. Conjunctiva demonstrated the highest infection rate (78% of samples infected [14/18]); however, infection rates did not differ statistically between cell types and viral isolates. After inoculation, 40% (4/10) of DSAEK grafts had active infection in the endothelium. SARS-CoV-2 lineage A demonstrated < 1 log decline in viral titers out to 14 days in corneal preservation media.

**Conclusions:**

SARS-CoV-2 lineage A variants can infect corneal, limbal, and conjunctival epithelium, as well as corneal endothelium. There was no statistical difference in infectivity between different lineage A variants. SARS-CoV-2 lineage A can survive and remain infectious in corneal preservation media out to 14 days in cold storage.

## Background

Coronavirus disease 2019 (COVID-19) is a life-threatening disease, spread primarily through respiratory droplets, that is caused by severe acute respiratory syndrome coronavirus 2 (SARS-CoV-2) [1]. Since its initial outbreak in Wuhan, China in December 2019, COVID-19 has become a prolonged global pandemic with a rising death toll [1, 2]. Based on genome sequencing of early SARS-CoV-2 samples, lineage A variants from China likely entered into the US through a single introduction event in Washington state in late January or early February of 2020 [3]. The virus then spread locally prior to the implementation of aggressive testing, resulting in the first wave. Since then, COVID-19 has continued to spread throughout the U.S. despite the availability of two mRNA-based vaccines and one viral vector-based vaccine since December of 2020 and February of 2021, respectively [4–6]. With ongoing logistical barriers hindering wide-spread global vaccination and the emergence of highly contagious delta and omicron variants, COVID-19 remains an major global health concern [7–11].

Although the primary clinical feature of COVID-19 is viral-associated pneumonitis, evidence suggests that the ocular surface may also be vulnerable to infection. Conjunctivitis has been a well-documented symptom of COVID-19, presenting in 0.8 to 32% of patients who contract the disease [12–14]. Additionally, viral RNA has been isolated from tears, conjunctival swabs, and other ocular tissues of COVID-19 positive patients with presumed lineage A variants [14–17]. Furthermore, in vitro studies have confirmed the presence of viral entry factors, angiotensin-converting enzyme 2 (ACE2) and transmembrane protease serine 2 (TMPRSS2), in both the conjunctival and corneal epithelium [18–20]. Due to this body of evidence, there is concern that the ocular surface may be susceptible to SARS-CoV-2 infection and COVID-19 patients may harbor infectious virus in their ocular tissues.

As such, there is also concern that corneas recovered from COVID-19 positive donors may be capable of transmitting disease to recipients after transplantation. Though donor-to-recipient transmission of COVID-19 following corneal transplantation has never been reported, donor-to-recipient transmission following lung transplantation has [21]. Additionally, experiments performed in rhesus monkeys have demonstrated that SARS-CoV-2 inoculation of the conjunctiva alone is sufficient to cause COVID-19-like interstitial pneumonia [22]. Furthermore, COVID-19 transmission through ocular exposure alone has been reported in at least one patient from China [23, 24]. As such, there is significant concern for donor-to-recipient COVID-19 transmission with transplantation of infected corneal tissue.

Even without disease transmission, transplantation of infected corneas may still pose a risk for corneal transplant recipients. Active viral replication and lysis of cells in donor endothelium may result in early graft failure due to endothelial cell loss. Furthermore, a host immunological response to retained viral proteins in the donor tissue may also be sufficient to trigger acute graft rejection in recipients [25]. Due to these concerns, the Eye Bank Association of America (EBAA) has issued rigorous guidelines to screen out COVID-19 donors [26].

Given the situation, viral infection studies are needed to determine whether transplantable ocular tissues can truly harbor infectious virus. Although in vitro infection of ocular tissues has previously been reported for limbal and corneal epithelium, the susceptibility of conjunctiva and endothelium to in vitro infection with SARS-CoV-2 remains unclear [27]. The infectivity of corneal endothelium is particularly important because the endothelium is often selectively transplanted alone in endothelial keratoplasty. Additionally, the stability of infectious SARS-CoV-2 in contaminated corneal preservation media has not been previously reported. To clarify the potential risks of using donor tissue from COVID-19 positive donors, further examination of the infectivity of donor tissue, particularly endothelium, is warranted. To that end, the purpose of this study was to test the susceptibility of transplantable ocular tissues to infection with SARS-CoV-2 lineage A variants using live viral infection studies; and, to evaluate the stability SARS-CoV-2 lineage A variants in standard donor cornea preservation media.

## Material and methods

Approval from the University of Minnesota Institutional Biosafety Committee (IBC) was obtained to perform in vitro infection studies using infectious SARS-CoV-2 virus. All handling of viral samples and infected tissue was performed in a certified biosafety level 3 (BSL-3) microbiology research laboratory. Human donor corneas and whole globes were obtained from Lions Gift of Sight (Minneapolis, MN, USA) with research consent under University of Minnesota Institutional Review Board (IRB) exemption for cadaveric tissue. All tissues used in this study were obtained from donors with negative COVID-19 testing within 3 days prior to death or no concerns for COIVD-19 infection based on EBAA/FDA screening criteria guidelines.

### Virus isolates and virus propagation

Two SARS-CoV-2 isolates, isolate 2019-nCoV/USA-WA1/2020 (NR-52281), and isolate hCoV-19/Hong Kong/VM20001061/2020 (NR-52282), deposited by the Centers for Disease Control and Prevention, were obtained through BEI Resources, NIAID, NIH (Manassas, VA). The USA-WA1 isolate was isolated from an oropharyngeal swab taken on January 2020 from a patient in Washington, USA who developed COVID-19 after recent travel to China. The Hong Kong isolate was isolated from a nasopharyngeal aspirate and throat swab taken on January 2020 from an adult male patient in Hong Kong. Both isolates were assigned lineage A and GISAID clade S [28].

Infection into Vero E6 cells (ATCC CRL-1586) was performed to confirm the infectivity of both viral isolates and to propagate the viruses. Briefly, Vero E6 cells were cultured and maintained in Dulbecco’s modified Eagle medium (DMEM) supplemented with 5% heat inactivated fetal bovine serum (FBS). Cells in 75cm [2] cell culture flasks were inoculated with each SARS-CoV-2 isolate at a multiplicity of infection (MOI) of 0.1 in 3 mL of DMEM and incubated at 37 °C/5% CO_2_ for 1 h. After 1 h adsorption period, 7 mL of DMEM supplemented with 5% FBS was added to the flasks and incubated at 37 °C/5% CO_2_ for 4 to 6 days. Cells were observed every day for cytopathic effect (CPE). When CPE reached 50–60%, the culture supernatant was harvested and clarified by centrifugation at 3,000 rpm for 15 min at 4 °C. Virus stocks were stored in aliquots at -80 °C. Virus titer was determined by 50% tissue culture infectious dose (TCID_50_) assay on Vero E6 cells. The infected Vero E6 cells were then fixed and immunostained for SARS-CoV-2 nucleocapsid (N)-protein and SARS-CoV-2 spike (S)-protein to confirm infection and to establish positive controls for the primary antibodies.

### In vitro infection of ocular surface epithelium

To test infection of ocular surface epithelium, cell cultures of limbal, corneal, and conjunctival epithelial cells were established from SARS-CoV2 negative donor tissue. After dissecting the limbal ring, bulbar conjunctiva, and central cornea from donor corneoscleral disks, tissue samples were segmented into ~ 1 × 1 mm tissue blocks and plated on plastic culture dishes for explant cultures as previously described [29, 30]. Explants were cultured for outgrowth at 37 °C/5% CO_2_ using EpiLife medium (Thermo Fisher Scientific, Waltham, MA, USA) with S7 supplement/penicillin/streptomycin for up to 14 days. Cells were then trypsinized and reseeded into 24-well tissue culture plates for in vitro infection studies.

Cultured cell lines were then each infected with both the US-WA1 or the Hong Kong SARS-CoV-2 isolates at an MOI of 0.5 to compare infectivity rates. At 2 days post infection (DPI), culture supernatant was removed, and the cells were washed twice with phosphate buffered saline (PBS). The cells were then fixed with 4% paraformaldehyde for 30 min at room temperature and immunostained for N-protein expression to detect viral infection.

### In vitro infection of endothelial cells

Infection of corneal endothelium was tested in flat-mounted Descemet’s Stripping Automated Endothelial Keratoplasty (DSAEK) grafts. DSAEK grafts were prepared from donor research corneas with transplant-quality endothelium. Grafts were cut to ≤150 μm thickness using a microkeratome (Moria SA, Antony, France) and trephined using a 7.75 mm corneal vacuum trephine (Katena, Parsippany, NJ) per standard protocols [31]. DSAEK grafts were placed into 6 well tissue culture plates and incubated with 8 × 10 [5] TCID_50_ of SARS-CoV-2 Hong Kong isolate in 1 mL of DMEM without FBS for 1 h at 37 °C/5% CO_2_. After 1 h adsorption period, grafts were transferred to 100 mm dishes containing 20 mL of DMEM/F12 supplemented with 10% FBS and incubated at 37 °C/5% CO_2_ for 4 days. Grafts were then fixed and immunostained for SARS-CoV-2 N-protein expression to detect viral infection.

### Immunostaining of cultured epithelial cells

Fixed cells were washed with PBS containing 0.3% Triton X-100 (PBST), blocked with 1% bovine serum albumin (BSA) and 2% normal goat serum in PBST for 1 h at room temperature and then incubated with rabbit anti-SARS-CoV-2 nucleocapsid antibody (SB40588-T62, Sino Biological; 1:2000 dilution) or anti-SARS-CoV2 spike antibody (SB40592-T62, Sino Biological, 1:500 dilution) overnight at 4 °C. After washing twice with PBST, cells were incubated with Alexa Fluor 594-conjugated goat anti-rabbit IgG (Life Technologies; 1:1000 dilution) at room temperature for 1 h. Cells were subsequently washed twice with PBST and treated with bis-Benzimide Hoechst 33258 (Sigma; 2 μg/mL) for 20 min at room temperature to counterstain the cell nuclei. After washing twice with PBST, cells were imaged using Nikon TE2000-E inverted fluorescent microscope.

### Immunostaining of DSAEK grafts

Virus-infected DSAEK grafts were fixed in 4% paraformaldehyde for 24 h and then permeated with 0.1% PBST. After extensive washing, non-specific immunoglobulin binding was blocked with 10% normal goat serum/1xPBS for 1 h at room temperature. The tissues were incubated overnight with the anti-SARS-CoV2 nucleocapsid antibody (1:500) followed by Alexa Fluor 594-conjugated goat anti-rabbit IgG (1:800). Cell nuclei were counterstained with Hoechst 33258 for visualization. All images were taken with a Leica DM 4000B fluorescence microscope using Leica Application Suite version 3.8 (Leica Microsystems, Buffalo Grove, IL).

### Virus viability in Life4°C medium

Corneoscleral discs were dissected from whole globes and placed in cornea viewing chambers (CVC) with 20 ml of Life4^o^C (Numedis Inc., Isanti, MN, USA) preservation medium for viral stability testing. Life4^o^C is a widely used cornea preservation media containing buffered minimal essential media, chondroitin sulfate, dextran 40, gentamicin, streptomycin, insulin, and other supplements [32]. Viability of infectious SARS-CoV-2 in Life4^o^C cornea storage medium was assessed by inoculating SARS-CoV-2 Hong Kong isolate into CVCs containing Life4^o^C with or without donor corneoscleral disks to a final concentration of 1 × 10 [5] TCID_50_/mL. The medium was stored at 4^o^C for 14 days. An aliquot of preservation medium was collected at 0, 1, 2, 4, 7, and 14 DPI and infectious virus titer were determined by TCID_50_ assay using Vero E6 cells.

### TCID_50_ assay

Vero E6 cells were seeded into 48-well cell culture plate 24 h prior to infection. Each aliquot sample of preservation medium were serially diluted eight times in DMEM using a 10-fold dilution each time. 100 μL of each dilution was then added to 48 well plates containing Vero E6 cells (6 wells for each dilution). Plates were incubated for 1 h at 37 °C/5% CO_2_. After 1 h incubation, 1 mL of DMEM supplemented with 2% FBS was added to each well and incubated for 48 h. Culture medium was then removed from the wells, cells were washed twice with PBS, and fixed with 4% paraformaldehyde for 30 min at room temperature. Wells were scored for presence or absence of viral protein immunostaining as described above. TCID_50_ was then calculated using Reed and Muench method based on the loss of positive immunostaining with serial dilution.

## Results

The USA-WA1 and Hong Kong SARS-CoV-2 isolates both showed extensive infection of Vero E6 cells at 2 DPI. Cells infected by both isolates demonstrated robust expression of SARS-CoV-2 N-protein and S-protein on immunostaining, providing a positive control for our primary antibodies.

### In vitro infection of ocular surface epithelium

**Fig. 1:**
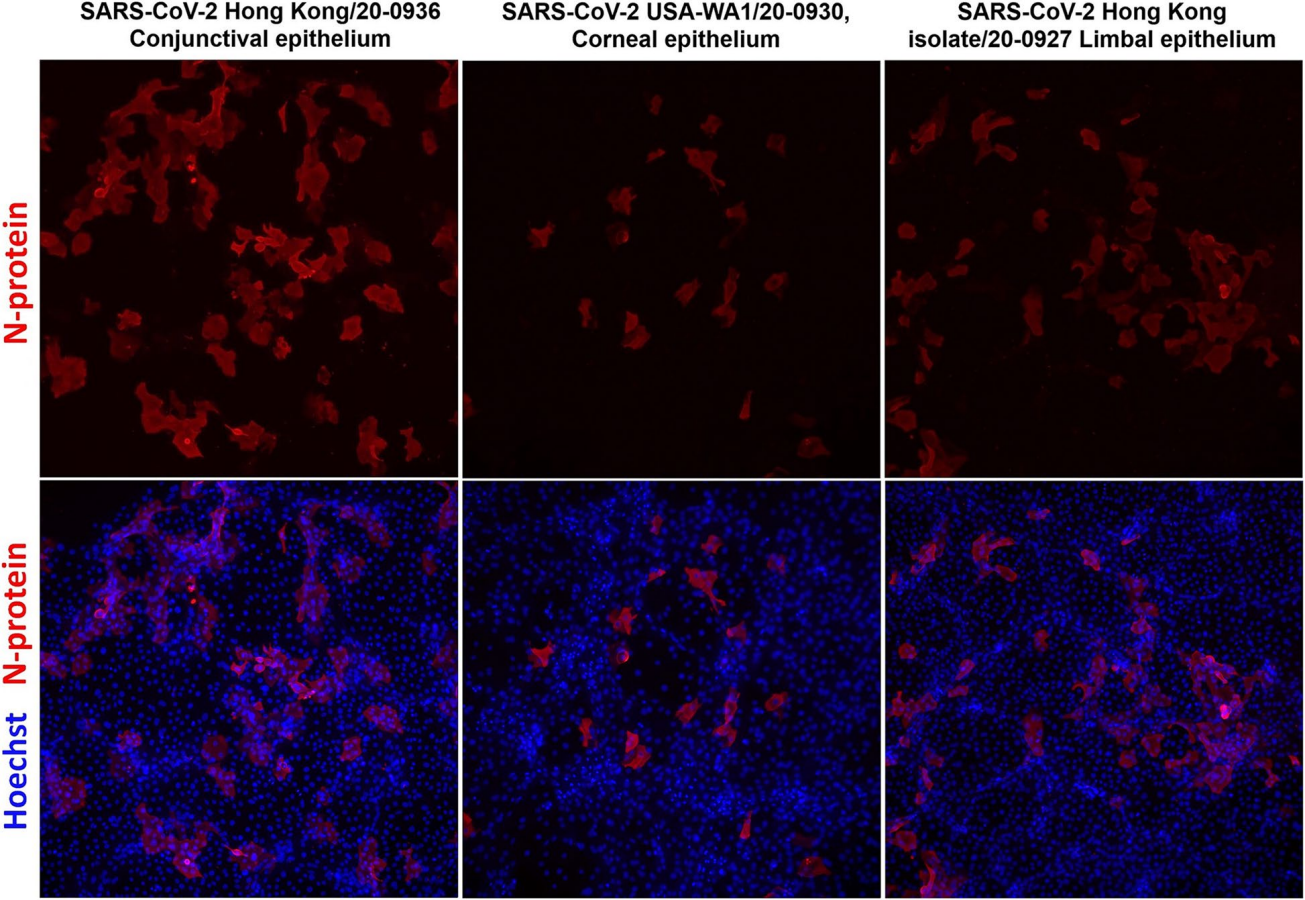
Immunohistochemistry demonstrating positive expression of SARS-CoV-2 nucleocapsid (N)-protein expression in clusters of infected cultured corneal, conjunctival, and limbal donor epithelium after inoculation with USA-WA1 or Hong Kong SARS-CoV-2 isolate. This demonstrates active infection and replication of the virus in these cell cultures

In total, 9 different sets of corneal, conjunctival, and limbal epithelial cells were cultured from 9 separate donor tissues. Mean donor age was 78.2 (range, 64 to 98), with 66.6% (6/9) male and 33.3% (3/9) female (Table 1). All three epithelial cell types showed some susceptibility to infection with both the USA-WA1 and Hong Kong SARS-CoV-2 isolates at 2 DPI (Fig. 1). Overall, 67% (16/24) of samples became infected after inoculation with the USA-WA1 isolate and 68% (17/25) of samples became infected after inoculation with the Hong Kong isolate. There was no statistically significant difference in rates of infection between the two lineage A viral isolates (chi-squared test, *p* = 0.92).

**Table 1:** Summary of SARS-CoV-2 epithelial culture infection results

Donor	Corneal Epithelium			Conjunctival Epithelium			Limbal Epithelium		
Age/Gender	USA-WA1	Hong Kong	Uninfected control	USA-WA1	Hong Kong	Uninfected control	USA-WA1	Hong Kong	Uninfected control
**64 / M**	**(+)**	**(−)**	**(−)**	**(+)**	**(+)**	**(−)**	**(+)**	**(+)**	**(−)**
**71 / F**	**(+)**	**(−)**	**(−)**	**(+)**	**(+)**	**(−)**	**(−)**	**(−)**	**ID**
**72 / M**	**(−)**	**(+)**	**(−)**	**(+)**	**(+)**	**(−)**	**(+)**	**ID**	**ID**
**73 / M**	**(+)**	**(+)**	**(−)**	**(+)**	**(+)**	**(−)**	**(+)**	**(+)**	**(−)**
**76 / M**	**(+)**	**(+)**	**(−)**	**(+)**	**(+)**	**(−)**	**(+)**	**(+)**	**(−)**
**79 / M**	**(+)**	**(+)**	**(−)**	**(−)**	**(−)**	**(−)**	**(−)**	**(−)**	**(−)**
**82 / F**	**ID**	**(−)**	**ID**	**(+)**	**(+)**	**(−)**	**ID**	**(+)**	**ID**
**89 / M**	**(−)**	**(+)**	**(−)**	**(−)**	**(+)**	**(−)**	**(−)**	**(−)**	**ID**
**98 / F**	**(+)**	**(−)**	**(−)**	**(−)**	**(+)**	**(−)**	**ID**	**ID**	**ID**
**% infected**	**75%**	**56%**	**0%**	**67%**	**89%**	**0%**	**57%**	**57%**	**0%**

(+): Positive for SARS-CoV-2 Nucleocapsid protein by immunostaining

(−): Negative for SARS-CoV-2 Nucleocapsid protein by immunostaining

ID: Indeterminant; insufficient cells in sample to perform infection study

Conjunctival epithelium had the highest rate of infection (78%, 14/18) followed by cornea epithelium (65%, 11/17), and limbal epithelium (57%, 8/14). However, there was no statistically significant difference in infection rates between cell types [cornea vs. conjunctiva (chi-squared test, *p* = 0.39), conjunctiva vs. limbus (chi-squared test, *p* = 0.21), and cornea vs. limbus (chi-squared test, *p* = 0.67)] (Table 1). Furthermore, there was no statistically significant difference in infection rates for each individual isolate between cell types.

While only 22% (2/9) of donors had positive infections in all associated tissue samples, no (0/9) donors were completely resistant to infection across all inoculated samples. There was no statistically significant difference in infection rates between samples cultured from male versus female donors (chi-squared test, *p* = 0.34). Additionally, there was no notable trend in infection rates based on donor age.

### In vitro infection of endothelial cells

**Fig. 2:**
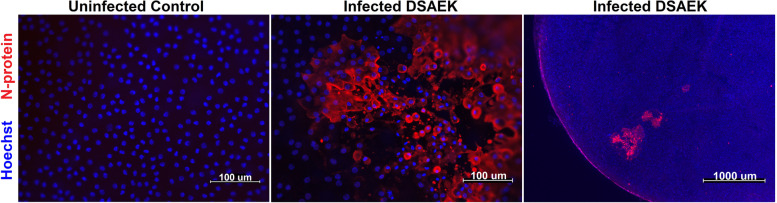
Immunohistochemistry demonstrating positive infection of the endothelium of a DSAEK graft after inoculation with the Hong Kong SARS-CoV-2 isolate. Multiple viral plaque are noted in the DSAEK graft on high (middle) and low magnification (left)

In total, ten DSAEK grafts were inoculated with the Hong Kong isolate. Out of ten DSAEK grafts inoculated, 40% (4/10) became infected with scattered viral plaque formation in the endothelium (Fig. 2). Two additional DSAEK grafts serving as uninfected controls showed no evidence of infection (Table 2). Mean endothelial cell density for the inoculated grafts was 2787 ± 462 cells/mm [2]. Mean donor age for inoculated grafts was 68 (range, 21 to 89). 50% (5/10) of the donors were male and 50% were female. There was no statistical difference in infection rate between male and female donors (chi-squared test, *p* = 0.20). No N-protein expression was noted in the posterior stroma or stromal keratocytes of any DSAEK graft samples.

**Table 2:** SARS-CoV-2 infection of DSAEK grafts

DSAEK sample #	Donor Age/Gender	Infection (Hong Kong strain)
**Control 1**	**66 / M**	**(−)**
**Control 2**	**92 / F**	**(−)**
**1**	**21 / F**	**(−)**
**2**	**43 / M**	**(−)**
**3**	**44 / M**	**(+)**
**4**	**60 / F**	**(−)**
**5**	**66 / M**	**(+)**
**6**	**67 / F**	**(−)**
**7**	**72 / M**	**(−)**
**8**	**74 / M**	**(+)**
**9**	**74 / F**	**(+)**
**10**	**89 / F**	**(−)**
**Infection rate**		**40%**

(+): Positive SARS-CoV-2 Nucleocapsid protein

(−): Negative SARS-CoV-2 Nucleocapsid protein

### Virus viability in Life4°C medium

**Fig. 3:**
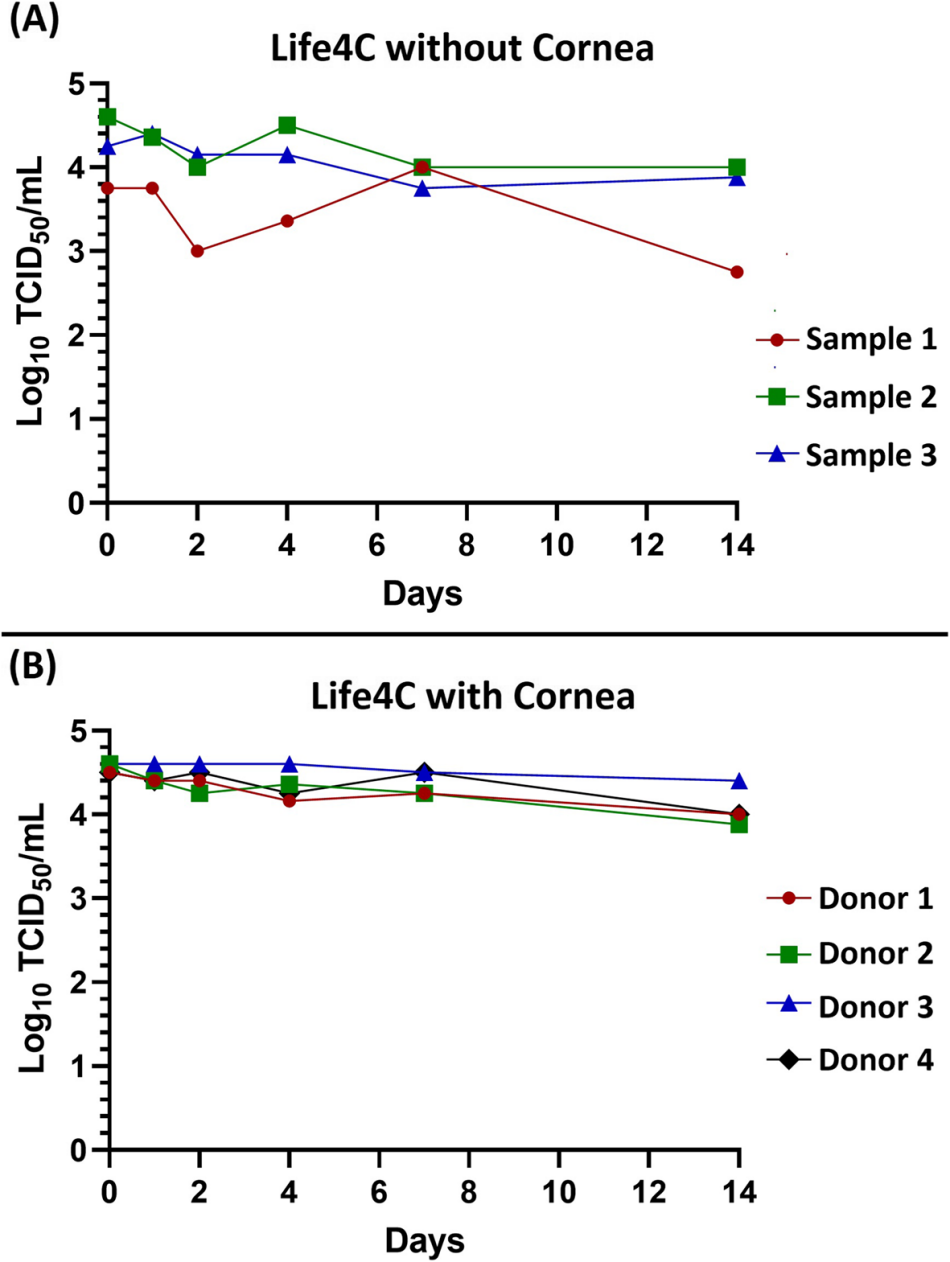
SARS-CoV-2 demonstrates a high degree of stability in Life4°C corneal preservation media with (**A**) or without (**B**) the presence of a donor. Under both conditions, infectious viral titers declined by less than a one log after 14 days in cold storage (4^o^C)

In three CVCs where Life4°C only (no cornea) was inoculated with the SARS-CoV-2 Hong Kong isolate, the viral titers (TCID_50_) remained relatively stable with less than a one log decline in any sample after 14 days in 4^o^C storage (Fig. 3A). Similarly, in four CVCs where Life4^o^C with a donor cornea was inoculated with the Hong Kong isolate, viral titers (TCID_50_) remained very stable with less than a 0.5 log decline (Fig. 3B). Overall, SARS-CoV-2 was found to be very stable in standard corneal preservation media, maintaining high levels of infectious viral titers out to 14 days.

## Discussion

COVID-19 remains an ongoing global pandemic and a major health concern despite the availability of three COVID-19 vaccines in the US. With the rapid spread of new SARS-CoV-2 variants, including the delta and omicron variants [11, 33, 34], potential transplantation of contaminated donor corneal tissue remains an ongoing concern for surgeons. To help clarify the risk of donor ocular tissues harboring infectious SARS-CoV-2 in transplantable ocular tissues, we performed live virus infection in ocular surface epithelium using lineage A variants. We further tested the infectivity of SARS-CoV-2 in pre-cut DSAEK grafts and evaluated the stability of infectious SARS-CoV-2 in standard cornea preservation media. Though previous studies have evaluated infectivity of SARS-CoV-2 in human corneal epithelium, limbal epithelium, and human embryonic stem cell (hESC)-derived eye organoids [27], this study further evaluates infection in all ocular surface epithelium (cornea, conjunctiva, and limbus), as well as in intact endothelial keratoplasty grafts. This study adds to the growing body of evidence around the risk of ocular infection and potential adverse events with transplantation of SARS-CoV-2 contaminated corneal tissue. Further evaluation of emerging variants is warranted to help refine public policy and donor screening guidelines based on risk of ocular disease transmission moving forward.

Overall, our study suggests that the corneal, conjunctival, and limbal epithelium are all susceptible to infection with SARS-CoV-2 lineage A variants. This is consistent with published evidence that has demonstrated that all these cell types express viral entry factors (ACE2, TMPRSS2) necessary for infection [17, 29]. Our results are also consistent with previous published in vitro studies in human corneal epithelium, limbal epithelium, and human embryonic stem cell (hESC)-derived eye organoids [27]. We also demonstrated that corneal endothelium is susceptible to infection based on in vitro infection of DSAEK grafts. This is of significant clinical relevance since endothelium is often selectively transplanted in corneal surgery. Given the susceptibility of the endothelium to SARS-CoV-2, even transplantation of contaminated DSAEK and DMEK may carry the risk of disease transmission. Furthermore, the formation of sizeable viral plaques in the endothelium of infected DSAEK grafts suggests that infected tissue may be at risk for early endothelial cell loss and graft failure, even in the absence of disease transmission.

It is interesting to note that no infection was observed in corneal keratocytes in any of the DSAEK grafts. This suggests that keratocytes are likely not susceptibility to SARS-CoV-2 infection. This finding is consistent with the limited expression of ACE2 and TMPRSS2 in corneal keratocytes which has previously been reported [29].

Given that conjunctival epithelium is easily cultured and high susceptible to infection, conjunctival epithelium may serve as a useful in vitro system for testing potential anti-viral agents. Such anti-viral agents may have a role as a supplement in corneal preservation media to sterilize contaminated donor corneas and further reduce the risk of transplanting contaminated tissue. Conjunctival epithelium may also be a relevant tissue for evaluating differences in infectivity and response to therapeutic drugs for emerging SARS-CoV-2 variants.

Though no correlation was found between donor characteristics (age, gender) and susceptibility of ocular tissues to infection, our study was limited by small sample size. A larger sample size using cohorts of donors with similar co-morbidities (i.e. diabetes, cardiac disease, etc), may be useful for elucidating donor risk factors that may increase the risk of SARS-CoV-2 infection.

SARS-CoV-2 demonstrated a high degree of stability in standard corneal preservation media (Life4^o^C) out to 14 days in cold storage. In comparison, SARS-CoV-2 has been shown to survive for less than 72 h on most surfaces, including plastic, stainless steel, copper, paper, and cardboard [35, 36]. This suggests that quarantining tissues even for the maximum allowable time (14 days) prior to transplantation is unlikely to be an effective method for decontaminating infected tissues. Standard corneal preservation media contain chondroitin sulfate, which helps stabilize the membrane of corneal endothelial cells [32, 37]. Since SARS-CoV-2 is an enveloped RNA virus [38], it is likely that the chondroitin sulfate also contributes to the stability of host cell derived SARS-CoV-2 envelopes in preservation media. The presence or absence of a donor corneoscleral disk did not significantly alter the stability of viral titers in the preservation medium. Active infection and viral replication in CVCs with a donor cornea may have been expected to result in an increase in viral titers, but the overall slight decline observed may be due to the presence of defensins or limited viral replication at 4^o^C.

In conclusion, the corneal endothelium, corneal epithelium, conjunctival epithelium, and limbal epithelium are all susceptible to SARS-CoV-2. Contaminated donor corneas may be at risk for early graft failure due to SARS-CoV-2 replication in the endothelium. The stability of SARS-CoV-2 in corneal preservation media precludes the use temporary quarantine measures to clear contaminated tissue. As such donor corneas should continue to be screened for COVID-19 as the best method to minimize the risk of transplanting contaminated tissue. Future testing of emerging variants, such as the delta and omicron variants, is warranted to confirm their ocular infectivity and stability in corneal preservation media.

## Data Availability

The datasets used and/or analyzed during the current study are available from the corresponding author on reasonable request.
